# The tetrapod fauna of the upper Permian Naobaogou Formation of China: 3. *Jiufengia jiai* gen. et sp. nov., a large akidnognathid therocephalian

**DOI:** 10.7717/peerj.6463

**Published:** 2019-02-22

**Authors:** Jun Liu, Fernando Abdala

**Affiliations:** 1 Key Laboratory of Vertebrate Evolution and Human Origins of Chinese Academy of Sciences, Institute of Vertebrate Paleontology and Paleoanthropology, Chinese Academy of Sciences, Beijing, China; 2 University of Chinese Academy of Sciences, Beijing, China; 3 CAS Center for Excellence in Life and Paleoenvironment, Beijing, China; 4 Evolutionary Studies Institute, University of the Witwatersrand, Johannesburg, South Africa; 5 Unidad Ejecutora Lillo (CONICET-Fundación Miguel Lillo), Tucuman, Argentina

**Keywords:** Akidnognathidae, Therocephalia, Upper Permian, Naobaogou Formation

## Abstract

Recent field trips to Member III of the Naobaogou Formation, Nei Mongol, China yielded new fossil discoveries, increasing our knowledge of the late Permian continental fauna from China. We present here a new large therocephalian, *Jiufengia jiai* gen. et sp. nov., represented by a partial skull with mandibles and part of the postcranial skeleton. This is the second therocephalian recovered from the Naobaogou faunal association and, in turn, the second akidnognathid from this unit and from China. The new taxon shows clear differences from *Shiguaignathus wangi*, the akidnogathid previously reported from the Naobaogou Formation: the presence of four upper postcanines, of a large suborbital vacuity, and the flat ventral surface of the vomer, lacking a ventromedian crest. Updating a previous phylogeny of therocephalians, we recover the new species as a basal member of Akidnognathidae, above a basal polytomy including the other two Laurasian akidnognathids, *Shiguaignathus* and *Annatherapsidus*, adding support to the hypothesis that this group originated in Laurasia.

## Introduction

The record of Chinese terrestrial strata of Permian age is well documented and has produced a good representation of amniotes (Li & Liu, 2015; Li, Wu & Zhang, 2008). In recent years, meticulous collective efforts in Permian localities have resulted in a remarkable expansion of our knowledge of the faunal members of terrestrial ecosystems (Li & Liu, 2013; Liu, 2013; Liu & Bever, 2015; Liu & Li, 2013; Liu et al., 2014; Reisz et al., 2011; Xu et al., 2015), and have also been successful in recording for the first time therocephalians in the Chinese Permian (Liu & Abdala, 2017a, 2017b). The Naobaogou Permian fauna was recently expanded by the presence of *Shiguaignathus wangi*, represented by a medium-sized and well-preserved snout, identified as the first Permian akidnognathid therocephalian from China (Liu & Abdala, 2017a), and of the pareiasaur *Elginia wuyongae*, which implied a significant expansion of the distribution of this parareptile (Liu & Bever, 2018).

Therocephalians constitute a heterogeneous group in the Permian, represented by three speciose lineages. One of these is Akidnognathidae, first erected as Akidognathinae by Nopcsa (1928) to include *Akidnognathus parvus* Haughton, 1918, represented by a small skull with a relatively broad snout from the *Cistecephalus* Assemblage Zone of South Africa, originally included in Scaloposauridae (Haughton, 1918). Later, authors considered Akidnognathidae to include *Akidnognathus*, *Cerdosuchoides*, *Scylacosaurus*, and *Ictidosaurus* (Haughton & Brink, 1954). The latter two genera are currently included in Scylacosauridae, a family including the basal and oldest therocephalians (Van Den Heever, 1994; Abdala, Rubidge & Van den Heever, 2008). The Family Euchambersidae (Boonstra, 1934; Haughton & Brink, 1954) was proposed to include the bizarre skull of *Euchambersia mirabilis* Broom 1931, although the spelling was later corrected as Euchambersiidae (Von Huene, 1940). Several other taxa considered now as akidnognathids were previously included in Moschorhinidae, Annatherapsididae (Mendrez, 1974a, 1975) and Euchambersiidae (Hopson & Barghusen, 1986). Nowadays Akidnognathidae, in a phylogenetic context, include the South African late Permian *Akidnognathus*, *Promoschorhynchus, Euchambersia*, and *Cerdosuchoides*, the Permo-Triassic *Moschorhinus,* the Early Triassic *Olivierosuchus*; the Russian late Permian *Annatherapsidus* and the recently described Chinese *Shiguaignathus* (Huttenlocker & Smith, 2017; Liu & Abdala, 2017a).

Here, we report the second akidnognathid from China and only the third for Laurasia, which clearly indicates that this predominantly Gondwanan lineage of therocephalian also has a reasonable representation in the Permian from Laurasia. In fact, two of the three therocephalian taxa reported for the Chinese Permian are members of the Akidnognathidae.

**Nomenclatural acts**. The electronic version of this article in portable document format will represent a published work according to the International Commission on Zoological Nomenclature (ICZN), and hence the new names contained in the electronic version are effectively published under that Code from the electronic edition alone. This published work and the nomenclatural acts it contains have been registered in ZooBank, the online registration system for the ICZN. The ZooBank Life Science Identifiers (LSIDs) can be resolved and the associated information viewed through any standard web browser by appending the LSID to the prefix http://zoobank.org/. The LSID for this publication is: urn:lsid:zoobank.org:act: EBA64D9C-595D-4AF7-B1CB-3B062623C4D2. The online version of this work is archived and available from the following digital repositories: PeerJ, PubMed Central, and CLOCKSS.

## Systematic Paleontology

THERAPSIDA Broom, 1905
THEROCEPHALIA Broom, 1903
EUTHEROCEPHALIA Hopson & Barghusen, 1986
AKIDNOGNATHIDAE Nopcsa, 1928
*Jiufengia jiai* gen. et. sp. nov.

**Etymology**. ‘Jiufeng’, refers to the name of the mountain where the fossil was collected; ‘Jia’ after Jia Zhen-Yan, the technician who discovered the specimen.

**Holotype**. IVPP V 23877, a skull with mandibles, incomplete right pectoral girdle, and incomplete right forelimb.

**Type Locality and Horizon**. Locality DQS 72, near Wuliangshitai, Gongshanwan, Tumd Right Banner, Nei Mongol, China; base of Member III, Naobaogou Formation.

**Diagnosis**. A large akidnognathid with the following autapomorphies: snout (preorbital region) longer than half of skull length; anteroposteriorly short temporal region with orbit being slightly larger than the temporal opening; jugal anterior process extends nearly to the level of the anterior margin of the lacrimal; ventral surface of vomer flat, lacking ventromedian crest; pterygoid transverse flange anterior to centre of orbit; prootic without central process; anteroventral process of the squamosal forms the bar anterior to the pterygo-paroccipital foramen; jugal tall below the orbit, intermediate between the condition of *Moschorhinus* and remaining akidnognathids.

## Description

**Figure 1:**
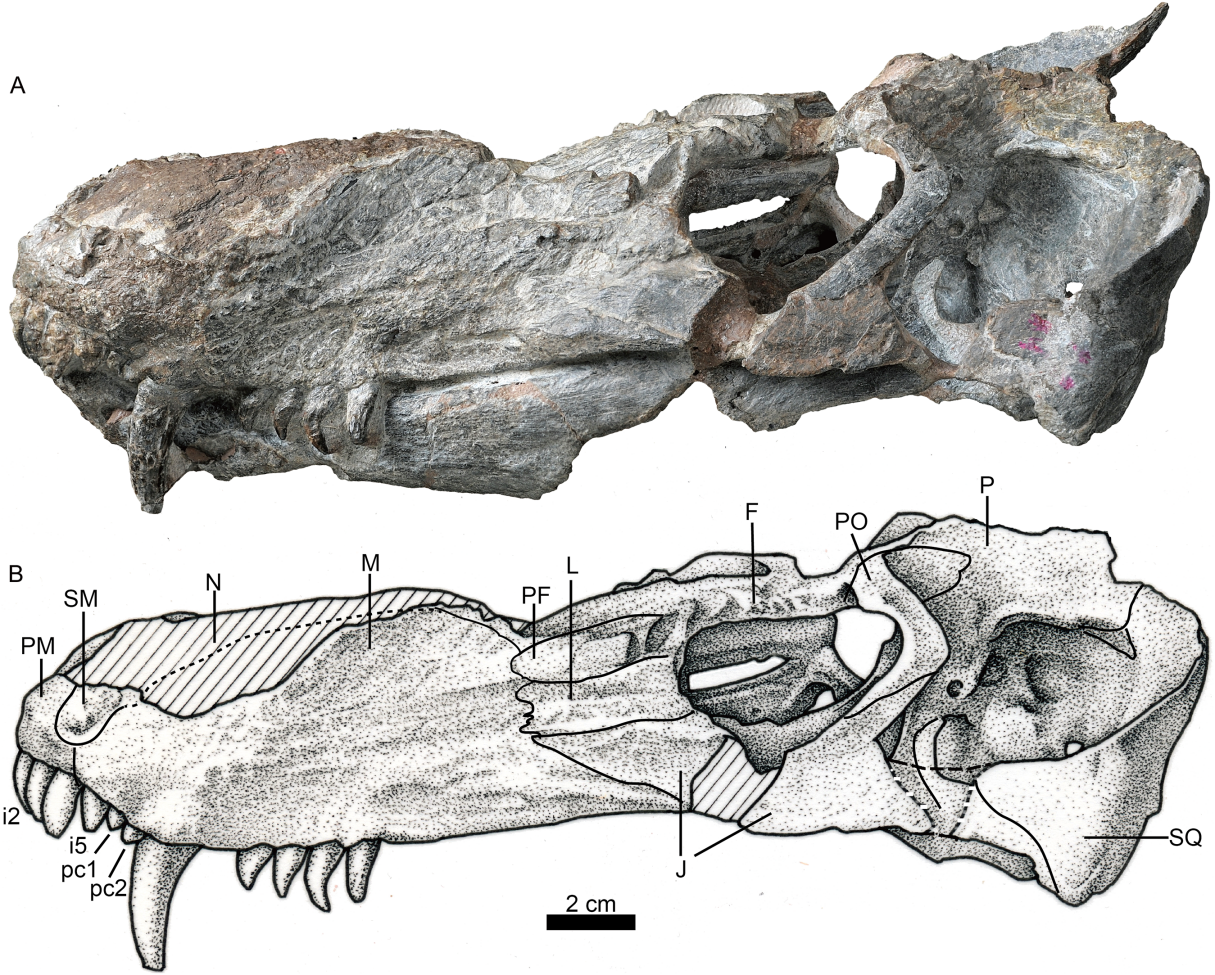
Holotype of *Jiufengia jiai* (IVPP V 23877) from the Naobaogou Formation of China. Photo (A) and line drawing (B) of the skull in left lateral view. Abbreviations: F, frontal; J, jugal; L, lacrimal; M, maxilla; N, nasal; P, parietal; PF, prefrontal; PM, premaxilla; PO, postorbital; SM, septomaxilla; SQ, squamosal. Photo credit: Wei Gao. Drawing credit: Yong Xu and Jun Liu.

**Figure 2:**
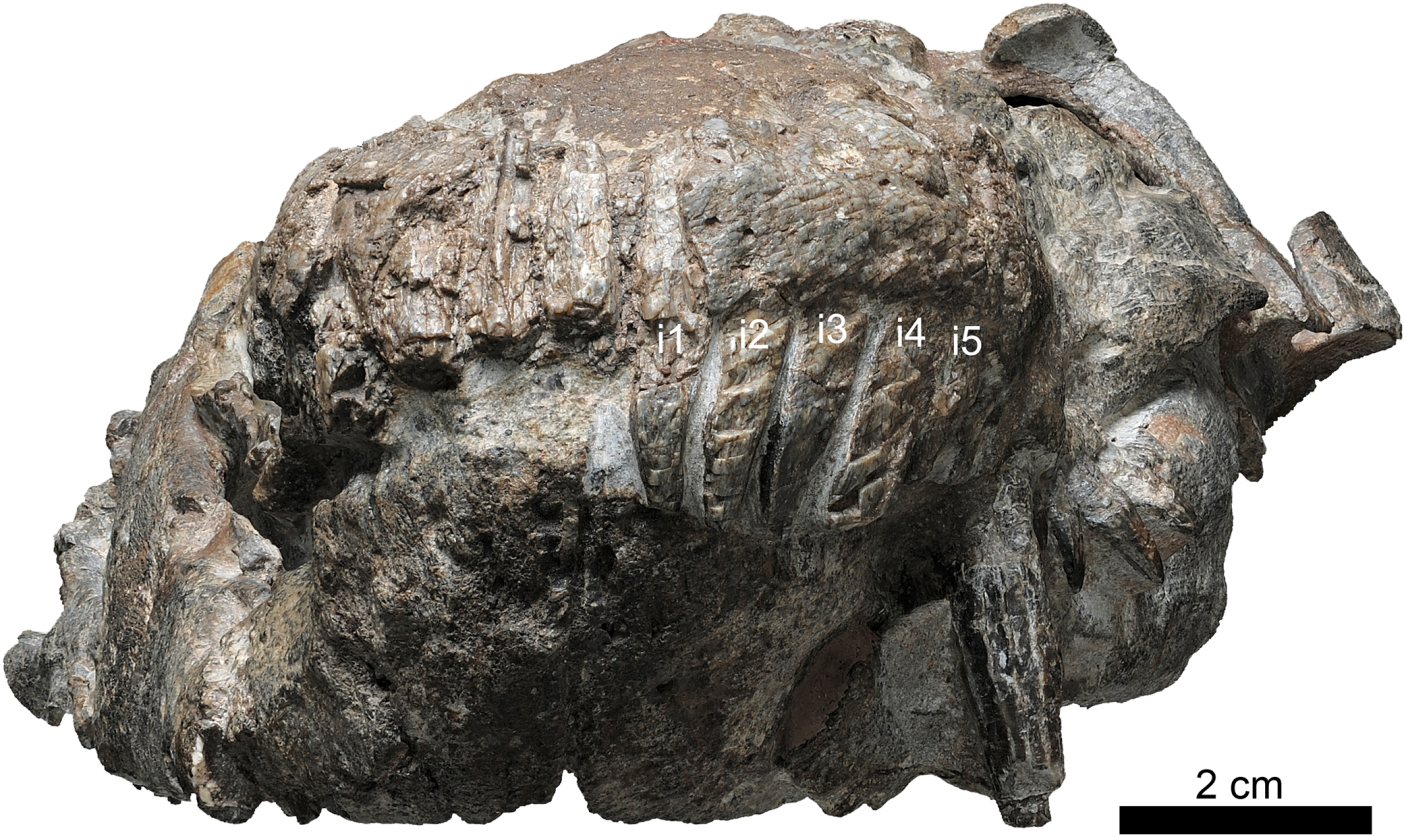
Holotype of *Jiufengia jiai* (IVPP V 23877) from the Naobaogou Formation of China. Photo of the skull and mandibles in anterior view. Abbreviation: I 1∼5, incisor 1∼5. Photo credit: Wei Gao.

**Figure 3:**
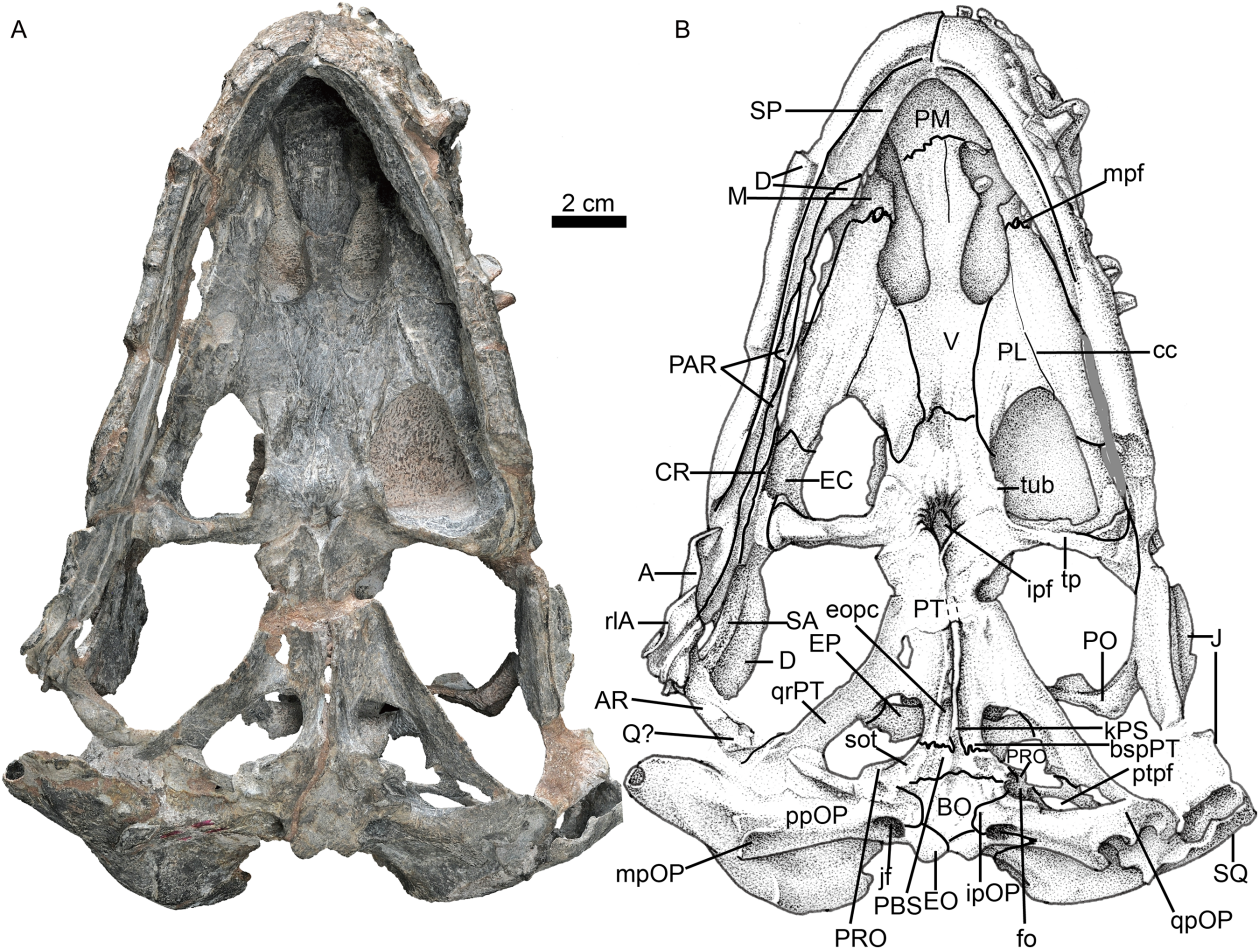
Holotype of *Jiufengia jiai* (IVPP V 23877) from the Naobaogou Formation of China. Photo (A) and line drawing (B) of the skull and mandibles in ventral view. Abbreviations: A, angular; AR, articular; BO, basioccipital; bspPT, basipterygoid process of the pterygoid; cc, crista choanalis; CR, coronoid; D, dentary; fo, fenestra ovalis; EC, ectopterygoid; EO, exoccipital; eopc, external opening of the parabasal canal; EP, epiptyerygoid; fo, fenestra ovalis; ipf, interpterygoid fossa; ipOP, internal process of the opisthotic; J, jugal; jf, jugular foramen; kPS, parasphenoid keel; M, maxilla; mpf, maxillo-palatine foramen; mpOP, mastoid process of the opisthotic; PAR, prearticular; PBS, parabasipterygoid; PL, palatine; PM, premaxilla; PO, postorbital; ppOP, paroccipital process of the opisthotic; PRO, prootic; PT, pterygoid; ptpf, pterygo-paroccipital foramen; Q, quadrate; qpOP, quadrate process of the opisthotic; qrPT, quadrate ramus of the pterygoid; rlA, reflected lamina of the angular; SA, surangular; sot, spheno-occipital tubercle; SP, splenial; SQ, squamosal; tp, transverse process; tub, tuberosity; V, vomer. Photo credit: Wei Gao. Drawing credit: Yong Xu and Jun Liu.

The specimen consists of the skull, mandibles, left scapula, right coracoid, partial right humerus, proximal portion of the right radius, and partial right manus. The skull is slightly crushed along the middle part of the dorsal surface, and some bones on its right lateral side and dorsal surfaces are eroded (Figs. 1–3). The skull occludes with the mandibles. A list of standard cranial measurements is provided in Table 1.

**Table 1:** Measurements of IVPP V 23877 (in mm).

Dorsal skull length, from tip of the snout to posterior margin of squamosal/occiput	250
Basal skull length from tip of the snout to occipital condyle	225
Maximum skull width	∼170
Snout (preorbital) length (left side)	145
Length from tip of the snout to anterior border of temporal fenestra	186
Breadth of rostrum at level of canines	68
Length of maxillary dentition	60
Total length of upper postcanine tooth row	32

### Skull roof

A prominent feature is the long snout, which represents more than half (58%) the length of the skull (Fig. 1; see Table 1). The skull has a triangular outline in dorsal view, being slightly constricted behind the canine and wider posteriorly. The general morphology resembles that of *Annatherapsidus*, but the latter does not show constriction behind the canine (Ivakhnenko, 2011: fig. 22a).

The laterally wide premaxilla features an eroded anterior tip and ascending processes. The anterior surface of the right side is also eroded exposing the roots of some incisors (Fig. 2). The premaxilla has a narrow exposure on the lateral side, covered by the anterior lamina of the maxilla (Fig. 1). The alveolar margin of the premaxilla is upturned anteriorly. The dorsal surface of the bone forms the ventral rim of the external nares with overlapping septomaxilla, only preserved on the left side. On the palate, the premaxilla meets the vomer with a short, trapezoidal, posteriorly-directed vomerine process (Fig. 3).

There are five upper incisors (Fig. 2). Although the lateral surfaces of the alveoli are eroded, the crowns of the left five incisors are still preserved, whereas the crowns have nearly vanished on the right side. The incisors are narrow, sharply conical, slightly curved lingually and somewhat anteriorly directed from alveoli. In lateral view, the alveolar margin is nearly straight. The anterior four incisors are similar in the crown height (ca. two cm), the fourth has the largest diameter, and the fifth is smaller.

**Figure 4:**
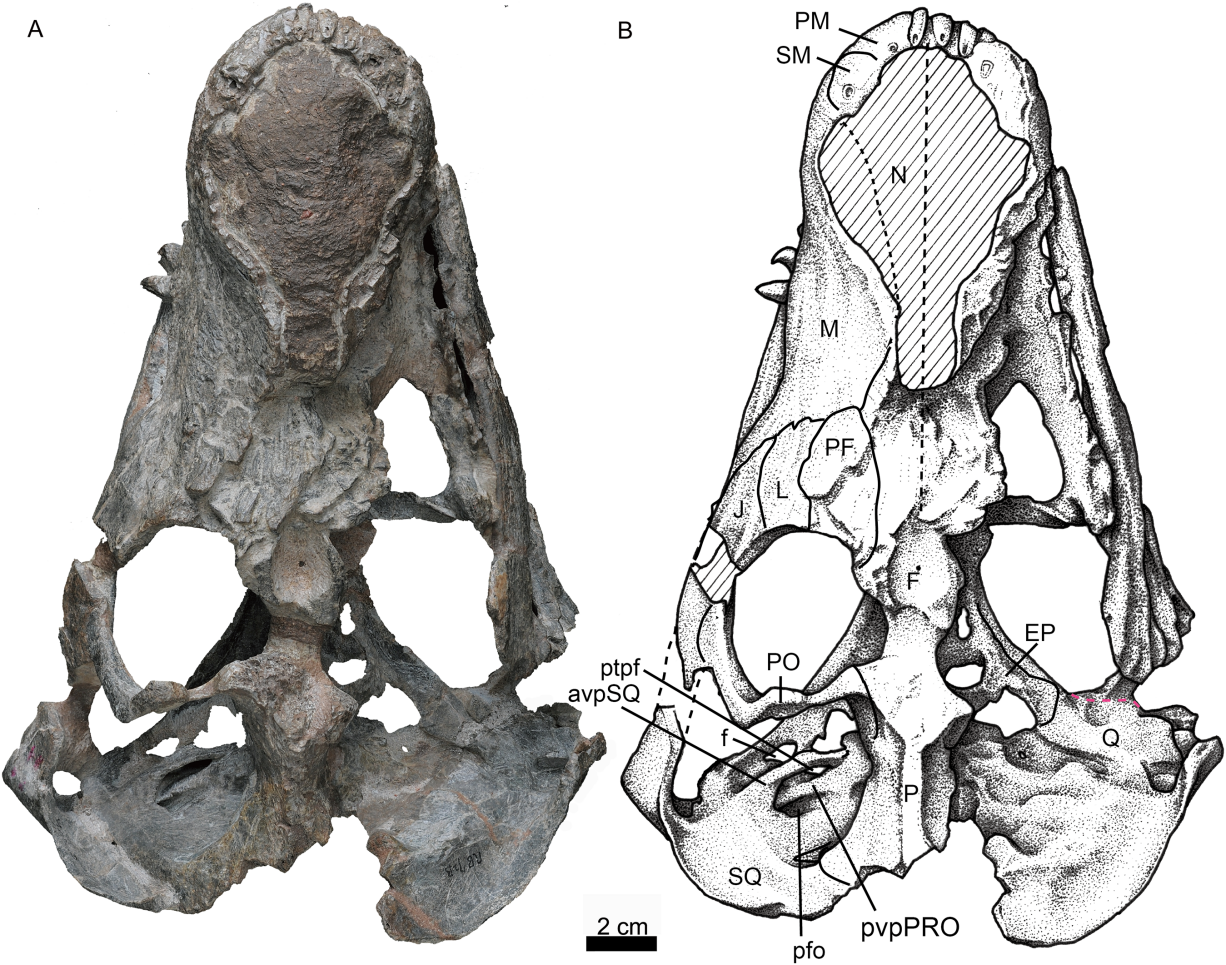
Holotype of *Jiufengia jiai* (IVPP V 23877) from the Naobaogou Formation of China. Photo (A) and line drawing (B) of the skull in dorsal view. Abbreviations: avpSQ, anteroventral process of the squamosal; EP, epiptyerygoid; F, frontal; f, foramen; J, jugal; L, lacrimal; M, maxilla; N, nasal; P, parietal; PF, prefrontal; PM, premaxilla; PO, postorbital; pfo, paroccipital fossa; ptpf, pterygo-paroccipital foramen; pvpPRO, posteroventral process of the prootic; Q, quadrate; SM, septomaxilla; SQ, squamosal. Photo credit: Wei Gao. Drawing credit: Yong Xu and Jun Liu.

The maxillary dorsal margin is incomplete, and most of the suture with the nasal is untraceable (Fig. 1). The maxilla contacts the prefrontal posterodorsally and the lacrimal and jugal posteriorly. It has a triangular posterior process which contacts the also triangular anterior process of the jugal. The maxilla is very high (height is close to 40% of the bone length) and long, forming the majority of the lateral region of the snout. It extends anteriorly to the position of the fourth incisor and contacts both the septomaxilla and premaxilla. Its height increases posteriorly, and the margins of the maxillae from both sides are separated a short distance on the dorsal surface (Fig. 4), suggesting the presence of a very narrow nasal at that point of the skull. The maxilla also bears numerous longitudinal grooves and pits, but not foramina, on the external surface of the facial plate. In lateral view, the ventral margin of the maxilla is slightly convex and directed anterodorsally in front of the level of the canine, while the posterior part is nearly straight.

In palatal view the maxilla has a broad exposure medial to the canine alveoli, approaching the wide anterior margin of the vomer. The maxillary exposure on the palate is constricted behind the canine, and its alveolar buccal margin is laterally concave. The maxilla houses two precanines, one canine, and four postcanines. The two small precanines are conical; the canine is cylindrical, with a diameter of 10 mm at the base. The apex of the canine is lost, and the preserved crown has more than three cm in height. The canine extends below the ventral margin of the lower jaw (Fig. 1). The canine is slightly curved posteriorly, and its crown surface is covered by regular longitudinal striations. A wide diastema of 8 mm is present posterior to the canine. The postcanines are conical, with a massive base and posteriorly curved crown, which is slightly flattened labiolingually. The two more posterior postcanines have bigger crowns than the first two. The third postcanine is more strongly curved posteriorly when compared with the other postcanines.

Only the posterior portion of the nasals is preserved, close to the prefrontals. The dorsal surface of the nasal on the left side is relatively well-preserved. The suture between the nasal and the frontal is unclear. If the posterior extension of the nasal is level with the anterior margin of the orbit, as in *Annatherapsidus*, the length of this bone is almost half of the skull length. No mid-sagittal crest is observed on the nasal.

Only the left orbit is preserved (Figs. 1 and 4). The orbit is rounded in dorsal view, and has a 44 mm anteroposterior length. The anterior wall of the orbit is formed by the prefrontal and lacrimal; the relatively deep suborbital bar is formed by the jugal, and maxilla only anteriorly; and the moderately slender postorbital arch is formed by the jugal and postorbital. The dorsal roof of the left orbit is incomplete. The lacrimal sutures with the prefrontal dorsally and the jugal ventrally. It is a rectangular bone in lateral view (Fig. 1). The triradiate jugal has a long anterior process, which reaches nearly the level of the anterior margin of the lacrimal. The jugal also extends anteriorly beyond the anterior margin of the orbit in *Akidnognathus* and *Annatherapsidus*, but it does not approach the anterior margin of the lacrimal (Ivakhnenko, 2011). Posteriorly, it contacts the anterior process of the squamosal approximately midway beneath the temporal fenestra, forming the zygomatic arch.

**Figure 5:**
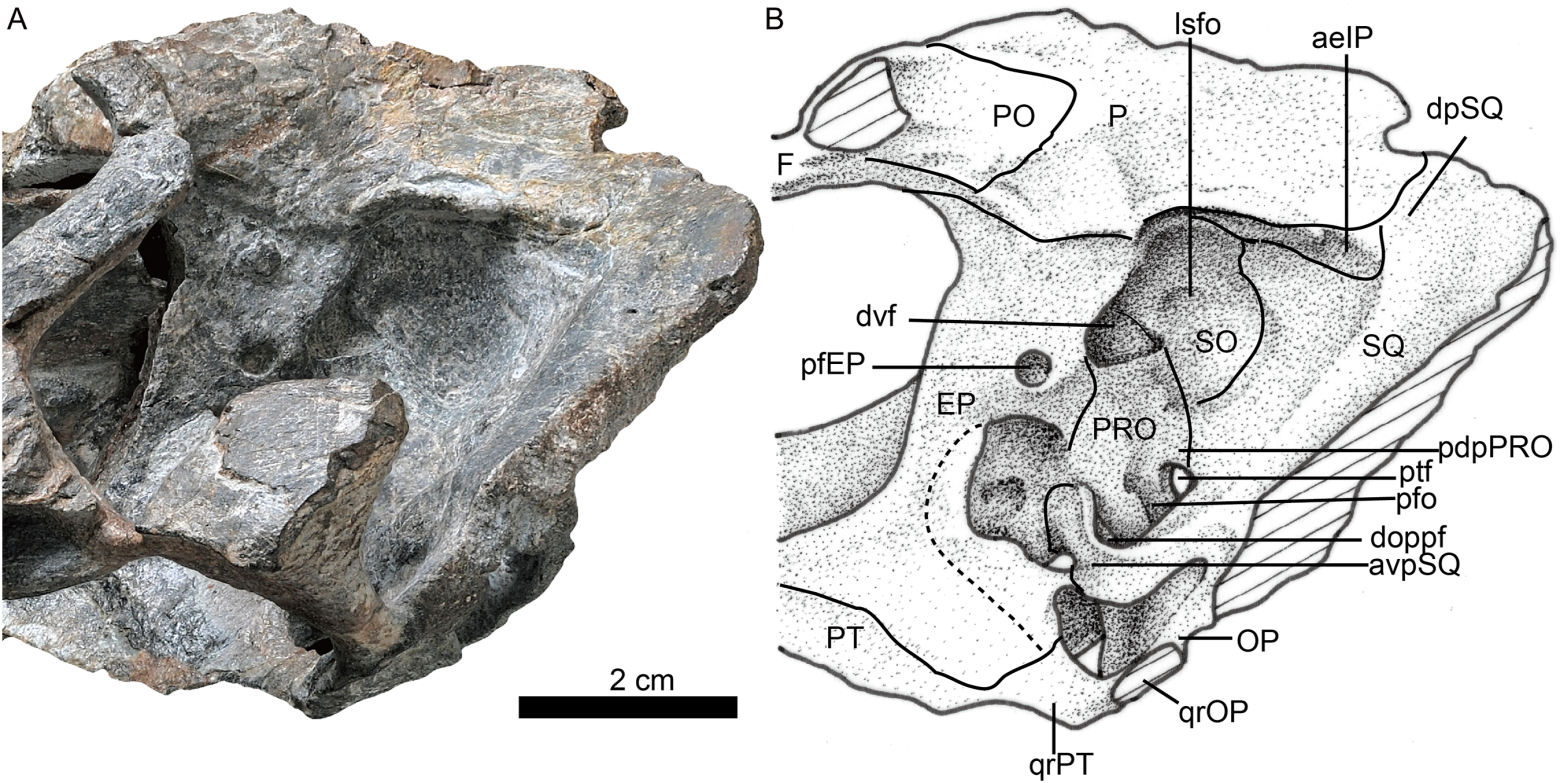
Holotype of *Jiufengia jiai* (IVPP V 23877) from the Naobaogou Formation of China. Photo (A) and line drawing (B) of the braincase in left lateral view. Abbreviations: aeIP, anterior extension of the interparietal; avpSQ, anteroventral process of the squamosal; doppf, dorsal opening of the paroccipital fossa; dpSQ, dorsal process of the squamosal; dvf, dorsal venous foramen; E, exoccipital; EP, epipterygoid; F, frontal; lsfo, lateral supraoccipital fossa; OP, opisthotic; P, parietal; pdpPRO, posterodorsal process of the prootic; pfEP, posterior foramen of the epipterygoid; pfo, paroccipital fossa; PO, postorbital; PRO, prootic; PT, pterygoid; ptf, post-temporal fenestra; qrOP, quadrate process of the opisthotic; qrPT, quadrate process of the pterygoid; SO, supraoccipital; SQ, squamosal. Photo credit: Wei Gao. Drawing credit: Yong Xu and Jun Liu.

The prefrontal is roughly triangular in dorsal view (Fig. 4). It contacts medially with the nasal and frontal. The frontal is poorly preserved. The postorbital has a long ventral process that covers the lateral surface of the jugal and partially forms the suborbital bar. The anterodorsal portion of the postorbital is missing, and the posterior process is short, forming the anterior lateral surface of the temporal fenestra, below the parietal crest (Fig. 5).

The temporal fenestra is roughly quadrangular (Figs. 1 and 4). It is only slightly longer than the orbit anteroposteriorly.

**Figure 6:**
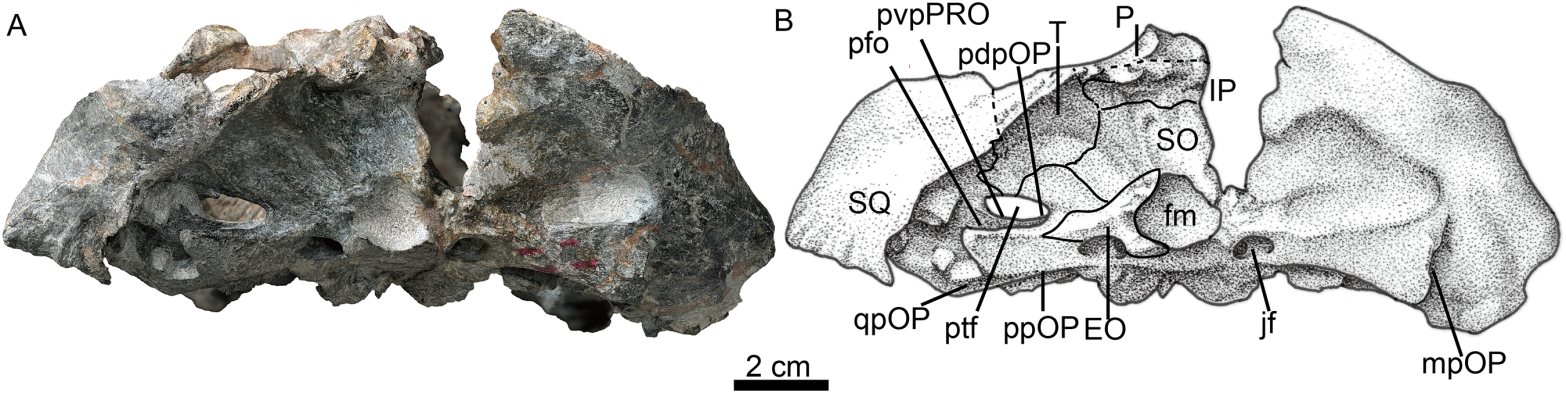
Holotype of *Jiufengia jiai* (IVPP V 23877) from the Naobaogou Formation of China. Photo (A) and line drawing (B) of the skull in occipital view. Abbreviations: EO, exoccipital; fm, foramen magnum; IP, interparietal; jf, jugular foramen; mpOP, mastoid process of the opisthotic; P, parietal; pdpOP, posterodorsal process of the opisthotic; ppOP, paroccipital process of the opisthotic; pvpPRO, posteroventral process of the prootic; qpOP, quadrate process of the opisthotic; SO, supraoccipital; SQ, squamosal; T, tabular. Photo credit: Wei Gao. Drawing credit: Yong Xu and Jun Liu.

The parietal is short, forming most of the narrow intertemporal region (Fig. 4). It forms a parietal crest which is more than half of the temporal fenestra length. There is no evidence of the parietal foramen due to poor preservation. The parietal forms the central portion of the lambdoidal crest, which is mostly eroded in the specimen, and also extends ventrally to contact the interparietal on the occiput (Fig. 6).

The squamosal extends medially to contact the parietal and posteriorly the tabular. In occipital view, it reaches the post-temporal fossa and forms its dorsolateral corner (Fig. 6). The squamosal extends ventrally to encase the lateral surface of the paroccipital process of the opisthotic, forming a rudimentary mastoid process (Fig. 3). The posteroventral margin of the squamosal forms a vertical notch to accommodate the quadrate and quadratojugal. Anteromedially, it has a long ventral process which contacts the quadrate process of the pterygoid and the paroccipital process. The squamosal sends an anteroventral process to contact the prootic medially and the epipterygoid anteroventrally (Figs. 4 and 5). This process forms the bar anterior to the pterygo-paroccipital foramen.

### Palate

The choana is confluent with the fossa for the lower canine, and it extends anteriorly to the level of the last incisor and posteriorly to the level of the third postcanine (Fig. 3). The choana is bordered laterally by the maxilla and palatine, anteriorly by the premaxilla, medially by the vomer, and posteriorly by the vomer and palatine.

The unpaired vomer is wide, anteriorly contacting the vomerine process of the premaxilla. The suture between these bones lies posterior to the level of the anterior margin of the choanae. Its anterior width is greater than half of the vomer length between choanae. The vomer is narrow at the level of the posterior margin of the choana, but it is much wider than in most akidnognathids, with the exception of *Moschorhinus* (Liu & Abdala, 2017a). The greatest width of the vomer is at the level of the posterior margin of the choanae, and progressively reduces its width posteriorly until it contacts the pterygoid, which is posterior to the level of the anterior margin of the suborbital vacuity, as in *Olivierosuchus parringtoni* (Botha-Brink & Modesto, 2011). The posterior plate of the vomer is ventrally concave, lacking a ventromedian crest. In most akidnognathids, the ventromedian crest is developed on both anterior and posterior plates of the vomer, and only on the posterior plate in *Olivierosuchus* (Botha-Brink & Modesto, 2011; Liu & Abdala, 2017a: fig. 6).

The palatine has a slightly curved medial suture with the vomer. Its lateral suture with the maxilla is almost parallel to the lateral margin of the skull (Fig. 3). The posterolateral process of the palatine extends to the middle of the lateral margin of the suborbital vacuity. The posteromedial side covers the pterygoid. The medial portion of the palatine almost lies on the same plane as the nearly flat lateral portion, and the posterior part of the crista choanalis is a crest on a nearly flat surface. The crista choanalis turns laterally behind the posterior margin of the choanae, and extends to the notch on the anterior margin of the suborbital vacuity. On the lateral portion, the palatine bifurcates in two short anterior processes located lateral and medial to the maxillo-palatine foramen. The maxillo-palatine foramen lies at the level of the anterior margin of the first postcanine.

The suborbital vacuity is large, slightly longer than it is wide (Fig. 3). The vacuities in the specimen have a different size, but the left one seems to be less distorted and closer to the natural size. The vacuity is formed anteriorly by the palatine, medially and posteriorly by the pterygoid, and posterolaterally by the ectopterygoid. On the medial margin of the vacuity the palatine and the pterygoid form two parasagittal crests, which become two tuberosities. Between them, a prominent ventromedial tuber lies anterior to the interpterygoid fossa, similar to the morphology observed in *Promoschorhynchus* (Mendrez, 1974b). The lateral lamina of the pterygoid is poorly developed.

The transverse process extends laterally and terminates in a swollen tuberosity (Fig. 3) as in *Annatherapsidus* (Ivakhnenko, 2011) and *Oliverosuchus* (Botha-Brink & Modesto, 2011). The transverse process is more or less horizontal on the medial side, but it turns nearly vertical laterally.

The ectopterygoid is contacted ventrally and anteriorly by the palatine, and it expands dorsoventrally at the posterior portion. The ectopterygoid forms a fossa on the posterolateral corner of the suborbital vacuity.

The interpterygoid fossa is quite small, as in *Annatherapsidus* (Ivakhnenko, 2011). It lies at the base of the transverse processes. The very short edges of the interpterygoid fossa meet to form a posteromedial crest, which connects with the high and sharp parasphenoid keel. The basisphenoid process of the pterygoid extends posteriorly to form the lateral margin of the external opening of the internal carotid canal, together with the parabasisphenoid. The lamina between the quadrate process and the basisphenoid process is strongly concave ventrally, bordered laterally by the strong crest of the quadrate process.

The dorsal portion, and possible partial ventral portion, of the right quadrate is preserved. It is an expanded lamina located adjacent to the anterior surface of the squamosal. There is no evidence of the quadratojugal.

### Braincase

The parabasisphenoid features an anterior parasphenoid keel and a wide, deep groove located between the strong spheno-occipital tubercles (Fig. 3). The parabasisphenoid forms the majority of the anterior margin of the fenestra ovalis, while the basioccipital forms the medial margin of the fenestra ovalis.

The posterior portion of the basicranium is incomplete with part of the basioccipital and exoccipitals eroded (Fig. 3). Anteriorly, the basioccipital participates in the formation of the spheno-occipital tubercle; laterally it contacts the opisthotic and posterolaterally it sutures with two exoccipitals, forming the ventral margin of the foramen magnum (Fig. 6).

The exoccipital forms the lateral portion of the occipital condyle and the foramen magnum (Fig. 3). The bone is nearly triangular in occipital view (Fig. 6). Its medial side forms the lateral wall of the foramen magnum, and its ventral side contributes to the roof of the large jugular foramen. The exoccipitals do not meet along the midline.

The occiput is inclined posteriorly (Fig. 3). In the occiput, the supraoccipital is fairly broad. It participates in the formation of the upper margins of the foramen magnum, separating the exoccipitals (Fig. 6). Anteriorly, it is also exposed in the temporal fossa (Fig. 5). From the margin of the foramen magnum, the supraoccipital has a ridge extending dorsolaterally that continues on the tabular, as in *Moschorhinus* (Durand, 1991). The area between these ridges has a deep indentation, mainly on the interparietal and the supraoccipital, for the attachment of the supravertebral cervical muscles. The supraoccipital has a short lateral extension between the opisthotic and the tabular.

The rectangular interparietal is a broad and low bone, with a short median ridge (Fig. 6). The high tabular forms a substantial portion of the lambdoid crest. It extends ventrally to contact the dorsolateral corner of the supraoccipital. Its ventral tip forms part of the dorsal border of the post-temporal fenestra.

The opisthotic consists of a robust paroccipital process, which forms the ventral border of the post-temporal fenestra in the occiput (Fig. 6). A shallow notch divides the mastoid process from the longer quadrate process (ventral flange). The mastoid process has only a rudimentary extension posteriorly, along with the posterior flange of the squamosal. A well-developed posterodorsal process of the opistothic contacts the tabular dorsolaterally and the supraoccipital dorsomedially, and forms half of the dorsal margin of the post-temporal fenestra. The internal process forms a lamina between the fenestra ovalis and the jugular foramen.

The left prootic is well preserved. Its basal area is in contact with the parabasisphenoid posteriorly and it forms the lateral side of the anterior border of the fenestra ovalis (Fig. 3). Its anteroventral and anterodorsal processes are not well exposed, being partially covered by the epipterygoid (Fig. 5). Dorsally, the prootic contacts the supraoccipital, and posterior to the epipterygoid there is a triangular incisure, interpreted as a venous foramen. The long posterodorsal and posteroventral processes contact the squamosal and form almost the entire dorsal and ventral margins of the posttemporal fenestra. There is no central process as described in *Promoschorhynchus* (Mendrez, 1974b). In ventral view, the suture between the prootic and the opisthotic runs medially towards the fenestra ovalis and continues on the dorsal border of the fenestra.

Both epipterygoids are preserved, but only the left one is complete (Figs. 3–5). It is a flat, blade-like bone (Fig. 5). It expands dorsally to contact the parietal, and its posterodorsal process contacts the supraoccipital posteriorly. It has a posterior apophysis that is posterior to the posterior foramen, which overlaps the anterodorsal process of the prootic. The basal part expands anteroposteriorly to cover nearly half the length of the quadrate ramus of the pterygoid. The posteroventral process contacts the squamosal, and a foramen (f in Fig. 4) is separated from the large cavum epiptericum.

### Mandible

**Figure 7:**
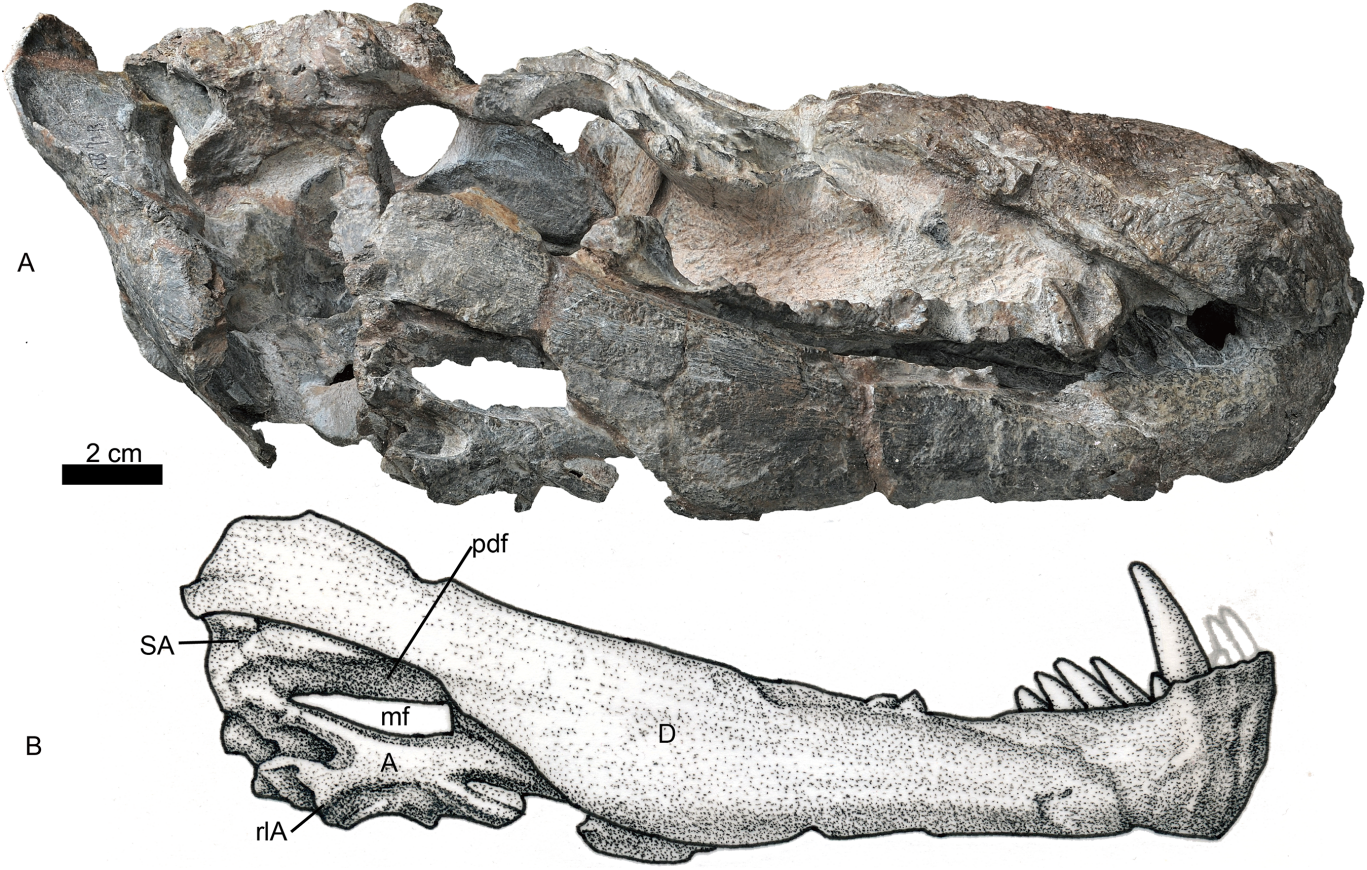
Holotype of *Jiufengia jiai* (IVPP V 23877) from the Naobaogou Formation of China. Photo (A) and line drawing (B) of the right mandible in lateral view. Abbreviations: A, angular; D, dentary; mf, mandibular fenestra; pdf, postdentary fossa; rlA, reflected lamina of the angular; SA, surangular. Photo credit: Wei Gao. Drawing credit: Yong Xu and Jun Liu.

Both mandibles are preserved, but the right one is more complete (Figs. 1 and 7). The dentary is long and deep, and the well-developed coronoid process has a straight terminal end. The horizontal ramus is constricted in height behind the canine, and distinctly expands posterior to the last postcanine (Fig. 7). A tall unfused symphysis forms a deep anterior chin. It is smoothly convex anteriorly in lateral view, forming an angulation of 110° with the dentary ventral margin. The dentary lateral surface is convex outwards, lacking any fossa. The dentary angle, anterolateral to the reflected lamina, is rounded and slightly convex.

The dentary contains four incisors, one canine and five or six postcanines. The fourth incisor is smaller and located lateral to the others based on the CT image. Five posteriorly inclined postcanines are counted on the left, and six on the right. However, the first right postcanine could be a replacing canine. The first left postcanine is smaller than the others, whereas the last right postcanine (four mm) is less than half the size of the previous teeth.

The splenial is an elongated bone (Fig. 3). Anteriorly, it meets its counterpart and participates in the formation of the lower part of the symphysis. It reaches the highest point below the second postcanine where its dorsal border is close to the upper margin of the dentary, and decreases in height from there until the level of the last postcanine, from where it extends posteriorly as a slender rod. It contacts the angular posteriorly behind the level of the transverse process, but the suture is not clearly visible. The ventral margin of the splenial is nearly confluent with that of the dentary near the symphysis, but it gradually rises posteriorly.

The postdentary fossa is elongated and formed by the surangular, prearticular, and angular (Fig. 7). The surangular is a narrow strip, having a concave ventral margin as the dorsal border of the mandibular fenestra. The mandibular fenestra is exposed laterally posterior to the dentary. The angular forms the ventral border of the fenestra on the lateral side. The angular reflected lamina has a wide ‘U’-shaped notch posteriorly, which is more posteriorly than dorsally directed. The lateral surface of the reflected lamina is ornamented with ridges, grooves and corrugations. The prearticular is a narrow, splint-like bone, which extends anteriorly to the level of the transverse process (Fig. 3). It forms the ventral margin of the mandibular fenestra on the medial side. A triangular coronoid forms the anterior margin of the mandibular fenestra on the medial side. The articular is partially preserved and has no features to mention.

### Postcranial skeleton

Some disarticulated postcranial bones were preserved with the skull. The following are the only identifiable elements.

**Figure 8:**
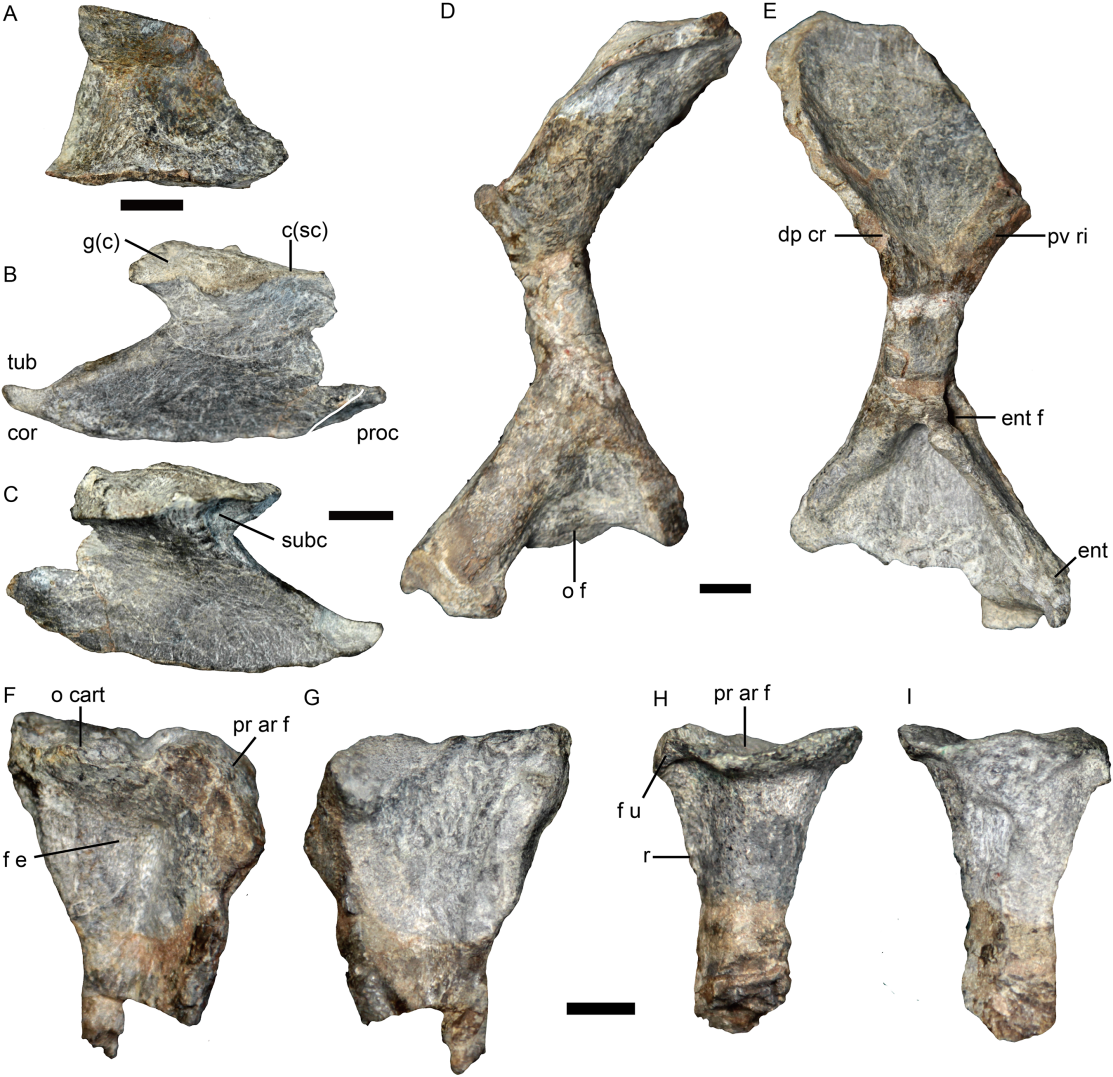
Holotype of *Jiufengia jiai* (IVPP V 23877) from the Naobaogou Formation of China. Photos of the postcranial bones. (A) Medial view of partial right scapula; (B) lateral and (C) medial views of right coracoid with small piece of procoracoid; (D) dorsal and (E) ventral views of right humerus; (F) lateral and (G) medial views of right ulna; (H) lateral and (I) medial views of right radius. Abbreviations: c(sc), coracoid articular surface for the scapula; dp.cr, deltopectoral crest; ent, entepicondyle; ent f, entepicondyle foramen; f e, fossa for extensor muscle origin; f u, facet for articulation with ulna; g(c), coracoid part of glenoid fossa; o cart, osseous base from which a presumably cartilaginous olecranon process arose; pr ar f, proximal articular facet; pv ri, posteroventral ridge; r, ridge; subc, fossa for subcracoideus; tub, tuberosity for origin of coracoid head of triceps. Scale bars equal one cm. Photo credit: Jun Liu.

**Pectoral girdle.** Only part of the ventral portion of the right scapula (Fig. 8A) was preserved. The right coracoid is complete, and a small piece of procoracoid is fused to it (Figs. 8B and 8C). The glenoid fossa seems to be formed by only the coracoid and scapula. The coracoid is expanded ventrally and curved inward toward the midline. The tuberosity for the coracoid head of the triceps lies posteroventral to the glenoid and far from it, as in *Promoschorhynchus* (Huttenlocker, Sidor & Smith, 2011) and other therocephalians (Kemp, 1986). A small fossa lies on the posterodorsal side of the medial surface of the coracoid, just below the articular surface. Some longitudinal striations are observed anterior to this fossa and might be for the attachment of the muscle subcoracoideus.

**Forelimb.** The right humerus lost most of its proximal and distal ends (Figs. 8D and 8E). The proximal half of the bone curves dorsally relative to the distal half. The proximal half is broad, with a smoothly concave ventral surface limited anteriorly by the deltopectoral crest and posteriorly by a ridge. The middle diaphysis is short and approximately circular in cross section, beyond which the bone expands to form the wide distal region. A large entepicondylar foramen lies in the posterior face and opens anteriorly into a deep trough on the ventral surface.

The proximal portions of the right ulna and radius are preserved. The ulna has no olecranon process, and the proximal side is rough (Figs. 8F and 8G). In anterior view, the proximal articular facet is lateromedially expanded. Ventral to the proximal end is a fossa for the origin of the extensor musculature (Fig. 8F). The radius has a thin shaft and expanded end. The proximal articulating facet is concave (Figs. 8H and 8I). A sharp ridge extends on the posterior side of the bone, below the facet for articulation to the ulna.

**Figure 9:**
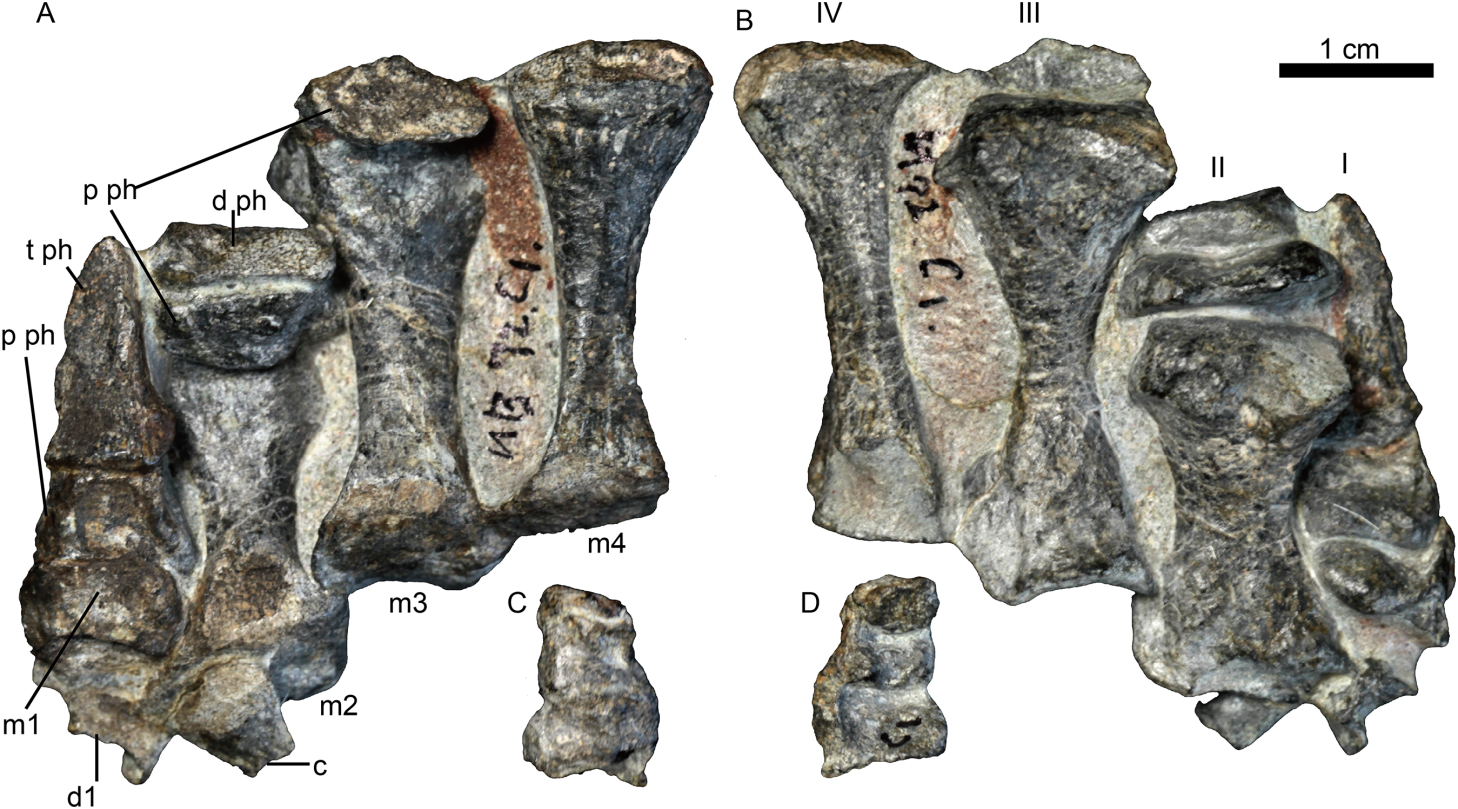
Holotype of *Jiufengnathus jiai* (IVPP V 23877) from the Naobaogou Formation of China. The right manus in (A) dorsal and (B) ventral views; three phalanges of digit IV in (C) dorsal and (D) ventral views. Abbreviations: d1, distal carpal 1; d2, distal carpal 2; d ph, distal phalange; m1-4, metacarpal 1-4; p ph, proximal phalange; t ph, terminal phalange. Photo credit: Jun Liu.

Elements of four digits of the right manus, identified as digits I–IV, are preserved together (Fig. 9). Their shape is similar to that of *Oliverosuchus* (Botha-Brink & Modesto, 2011). Distal carpals 1 and 2 are preserved. The medial surface of distal carpal 1 is strongly concave and the distal is articulated with the metacarpal I. A bone in contact with metacarpal II is interpreted as an out of place distal carpal 2. The bone is relatively large and transversely expanded on one side. Metacarpals I–IV are nearly complete, measuring 7, 22, 29, and 30 mm, respectively. The rectangular, wider than longer, metacarpal 1 is remarkably short and squat, differing from the quadrangular element of *Olivierosuchus* (Botha-Brink & Modesto, 2011: text-fig. 7). The other three metacarpals are long with expanded ends, and with similar length as the III and the IV, which are longer than the II. A similar pattern of metacarpal lengths is represented in *Olivierosuchus* (Fontanarrosa et al., in press: fig. 3B). The diaphysis of metacarpal II is remarkably wider than that of metacarpals III and IV (Fig. 9B). The phalanges preserved for each digit are 2-2-1-3, including three disarticulated phalanges from digit IV (Figs. 9C and 9D). The terminal (ungual) phalanx is narrow, pointed and claw-like, while the others are short, broad and squat.

## Discussion

The fossil record of therocephalians has been historically poorly represented in the extensive exposures of the terrestrial Chinese Permian. However, the recent discovery of two new, definitely late Permian therocephalians (Liu & Abdala, 2017a, 2017b) is challenging this situation. The akidnognathid *S. wangi* was the first therocephalian reported for the faunal association of the Naobaogou Formation. IVPP V 23877, here described as *J. jiai*, is the second therocephalian and, in turn, the second akidnognathid of the Naobaogou assemblage according to the following features: a septomaxilla well exposed outside of the external naris, broadly overlapping the premaxilla anteriorly; a very expanded vomer, broadly overlapping the vomerine process of the premaxilla; and contribution of the premaxilla and maxilla to a fossa for the lower canine on the palatal surface.

*Jiufengia jiai* differs from the smaller *S. wangi* from the same horizon in the presence of four upper postcanines, presence of a large suborbital vacuity, and ventral surface of vomer flat, lacking a ventromedian crest.

The dental formula of *Jiufengia* is I5:pC2:C1:Pc4/i4:c1:pc5. Only *Moschorhinus* and *Olivierosuchus* (with three; Durand, 1991; Botha-Brink & Modesto, 2011); and *Euchambersia* (with none; Benoit, 2016) have fewer postcanines than *Jiufengia*. Other akidnognathids have from five (e.g. *Promoschorhynchus*) to a maximum of eight recorded in the other Chinese Permian akidnognathid, *Shiguaignathus* (Liu & Abdala, 2017b). *Jiufengia* can also be differentiated from other akidnognathids by the long snout which is more than half the skull length, the transverse process is anterior to the orbit, the ventral surface of the vomer is flat along the middle line, lacking a ventromedian crest, and the anterior process of the jugal is nearly level with the anterior margin of the lacrimal. All these characters show that this specimen represents a new species within Akidnognathidae.

The suborbital vacuity of *J. jiai* is more similar to *Annatherapsidus* than other taxa in its large size, anterioposterior length greater than the width, long and relatively straight lateral margin which is more anteriorly than medially directed, and the posterior margin nearly laterally directed.

**Figure 10:**
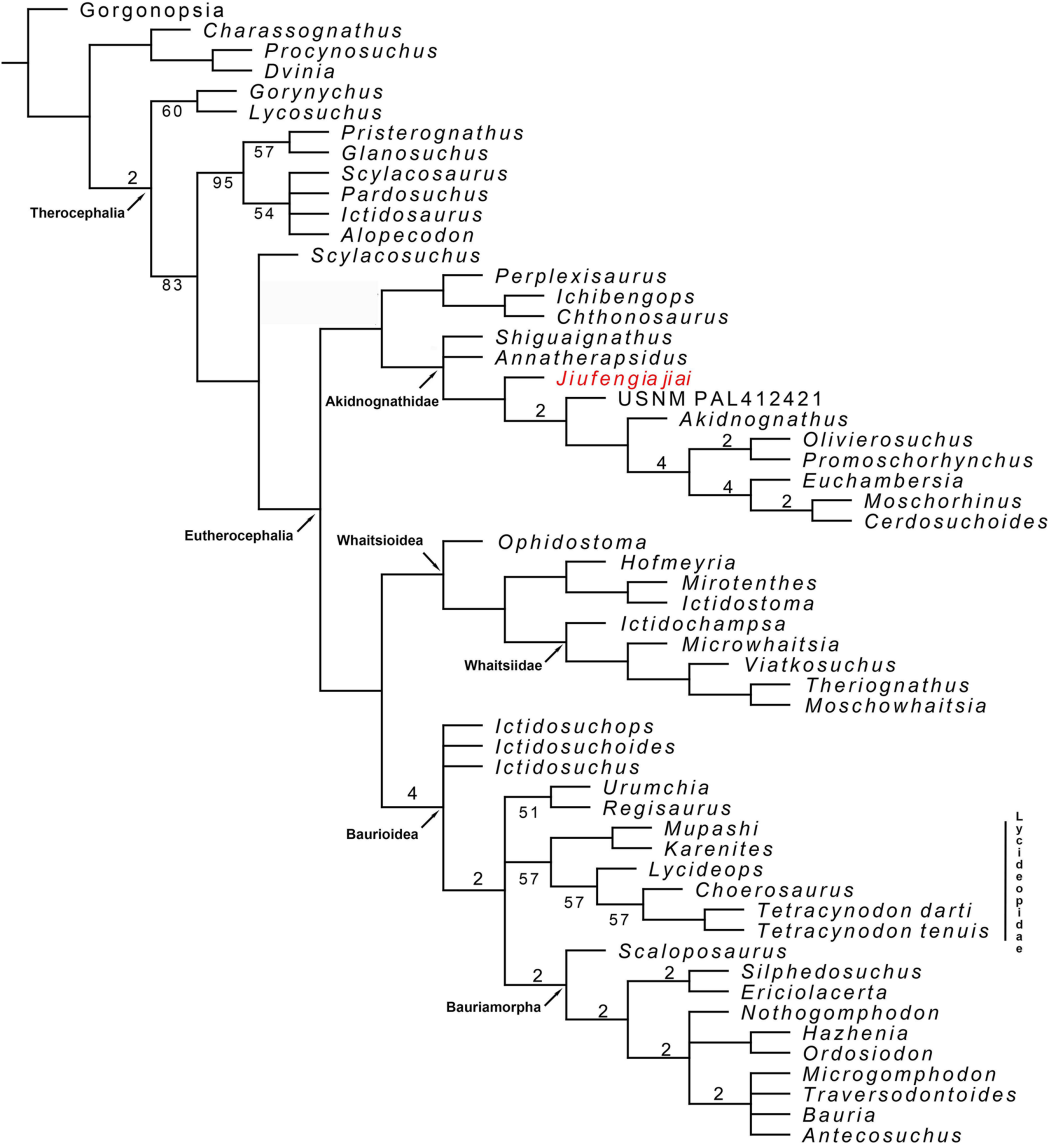
Majority consensus trees of Therocephalia relationships. *Jiufengia jiai* is indicated in red. Numbers below the branch indicates frequency of clades in the fundamental trees; numbers above the branch indicate Bremer support of the groups (only values of two or more are indicated).

To reconstruct the phylogenetic position of *J. jiai*, we coded it in our previous matrix (Liu & Abdala, 2017a) and included as a terminal the recently described Russian therocephalian *Gorynychus* (Kammerer & Masyutin, 2018). The matrix was analysed with TNT 1.5 (Goloboff & Catalano, 2016), as in Liu & Abdala (2017a): the search for most parsimonious trees (mpt) consisted of 10 random addition sequences and TBR, saving 10 trees per replication, and a second search using the trees from RAM as a starting point and implementing TBR on those trees. A total of 17 multistate characters were considered as additives. The search resulted in 7,560 mpt of 384 steps in which most of the major groups of Therocephalia are recovered as monophyletic (Fig. 10). The result is nearly identical to our previous analysis (compare the consensus tree in figure 10 with the right one in figure 7 of Liu & Abdala, 2017a). The only differences to report are in the placement of the two new terminals added in the current study. *Jiufengia* is recovered as a basal akidnognathid, following a polytomy at the base of this clade that includes the other two Laurasian akidnognathids *Shiguaignathus* and *Annatherapsidus* (Fig. 10). Apart from the Laurasian most basal records of Akidnognathidae, the current evidence shows most Laurasian akidnognathids were of medium-to-large size. Of the four late Permian akidnognathids in the Karoo Basin, only *Moschorhinus* at the end of the Permian shows a large size (maximum skull length 262 mm; Huttenlocker & Botha-Brink, 2013), with remaining taxa ranging between 107 and ∼135 mm in skull length (Table 2). The youngest record of akidnognathid in the Karoo, represented by *Olivierosuchus*, is one of the smaller representative of the lineage. In contrast, two of the three Laurasian akidnognathids show large sizes (above 200 mm, Table 2), and only *Shiguaignathus* is a medium-sized animal.

**Table 2:** Teeth count and skull length of akidnognathid specimens.

	Incisors	Precanines	Canine	Postcanines	Specimen	Max skull length (mm)
*Promoschorhynchus*	5	1	1	5	BP/1/484	
*Promoschorhynchus*	5	2	1	5	SAM-PK-K10014	123
*Promoschorhynchus*	5	1	1	5	RC 116	∼135
*Olivierosuchus*	5	2.1	1	3	BP/1/3849	102
*Moschorhinus*	5	1	1	3	NHMUK R5698	
*Moschorhinus*	5		1	?	BP/1/1713	262
*Annatherapsidus*	5	2?	1	6	Ivakhnenko (2011)	>200
*Akidnognathus*	5	1	1	7	Brink (1961)	
*Akidnognathus*	5	1	1	?7	SAM-PK-4021	107
*Euchambersia*	5		1		BP/1/4009	120
*Shiguignathus*	5		1	8	IVPP V 23297	
*Jiufengia*	5	2	1	4	IVPP V 23877	250

**Note:**

?: unsure.

Although the current matrix is only different from that of Kammerer & Masyutin (2018) in a few characters, the result is quite different regarding the placement of the Russian *Gorynychus* and *Perplexisaurus*. In our analysis *Gorynychus* is one of the basalmost therocephalians, recovered as a sister taxon of *Lycosuchus* (although only in the majority consensus tree; Fig. 10). Two characters support this monophyly: a deep suborbital bar and five or less upper postcanines in adults. *Perplexisaurus* is recovered as a basal member of the recently defined clade Chthonosauridae (Huttenlocker & Sidor, 2016). This group has only one synapomorphy: the parietal crest in adults extends forward to include parietal foramen. However, the score of this character is unknown in *Ichibengops*, therefore, the only synapomorphy of the group is ambiguous.

The differences between our result (that reflect mostly the hypothesis of Huttenlocker et al.) and that of Kammerer & Masyutin (2018) show instability in current hypotheses of therocephalian relationships, which is evident in the support values of monophyletic groups represented in Fig. 10. Thus, support values are overall low and are especially poor in basal Therocephalia, Eutherocephalia, Chthonosauridae, basal Akidnognathidae, Whaitsioidea, Whaitsiidae, and Lycideopidae.

## Supplemental Information

10.7717/peerj.6463/supp-1 Supplemental Information 1 Matrix used for the current analysis.

## Additional Information and Declarations

### New Species Registration

The following information was supplied regarding the registration of a newly described species: Publication LSID: urn:lsid:zoobank.org:pub:26E8108A-1D53-475C-A318-682C1B36D38D. Jiufengia LSID: urn:lsid:zoobank.org:act:EBA64D9C-595D-4AF7-B1CB-3B062623C4D2. *Jiufengia jiai* LSID: urn:lsid:zoobank.org:act:1DE90432-86D0-4CA3-A35E-FDE0B607A57A.

### Data Availability

The following information was supplied regarding data availability: The raw measurements are available in a Supplemental File. The raw data show the character codings.
